# The endemic *Helicobacter pylori* population in Southern Vietnam has both South East Asian and European origins

**DOI:** 10.1186/s13099-021-00452-2

**Published:** 2021-09-30

**Authors:** Trang Hoa Nguyen, Trang Thi My Ho, Thien-Phuc Nguyen-Hoang, Shamsul Qumar, Thuc Tran Dang Pham, Quy Nhuan Bui, Dieter Bulach, Thuy-Vy Nguyen, Motiur Rahman

**Affiliations:** 1 Oxford University Clinical Research Unit, 764 Vo Van Kiet Street, Ward 1, District 5, Ho Chi Minh City, Vietnam; 2 Department of Genetics, Faculty of Biology and Biotechnology, Ho Chi Minh University of Science, Ho Chi Minh City, Vietnam; 3 GeneStore India Pvt Ltd, Gurgaon, Haryana India; 4 Department of Gastroenterology, Gia Dinh Hospital, Ho Chi Minh City, Vietnam; 5 Melbourne Bioinformatics, The University of Melbourne and Doherty Applied Microbial Genomics, The Doherty Institute, Melbourne, Australia; 6 Centre for Tropical Medicine and Global Health, Nuffield Department of Medicine, Oxford University, Oxford, UK

**Keywords:** *H. pylori*, Vietnam, Molecular epidemiology

## Abstract

**Background:**

The burden of *Helicobacter pylori*-induced gastric cancer varies based on predominant *H. pylori* population in various geographical regions. Vietnam is a high *H.* *pylori* burden country with the highest age-standardized incidence rate of gastric cancer (16.3 cases/100,000 for both sexes) in Southeast Asia, despite this data on the *H. pylori* population is scanty. We examined the global context of the endemic *H. pylori* population in Vietnam and present a contextual and comparative genomics analysis of 83 *H. pylori* isolates from patients in Vietnam.

**Results:**

There are at least two major *H. pylori* populations are circulating in symptomatic Vietnamese patients. The majority of the isolates (~ 80%, 66/83) belong to the hspEastAsia and the remaining belong to hpEurope population (~ 20%, 17/83). In total, 66 isolates (66/83) were *cagA* positive, 64 were hspEastAsia isolates and two were hpEurope isolates*.* Examination of the second repeat region revealed that most of the *cagA* genes were ABD type (63/66; 61 were hspEastAsia isolates and two were hpEurope isolates). The remaining three isolates (all from hspEastAsia isolates) were ABC or ABCC types. We also detected that 4.5% (3/66) *cagA* gene from hspEastAsia isolates contained EPIYA-like sequences, ESIYA at EPIYA-B segments. Analysis of the *vacA* allelic type revealed 98.8% (82/83) and 41% (34/83) of the strains harboured the s1 and m1 allelic variant, respectively; 34/83 carried both s1m1 alleles. The most frequent genotypes among the *cagA* positive isolates were *vacA* s1m1/*cagA* + and *vacA* s1m2/*cagA* + , accounting for 51.5% (34/66) and 48.5% (32/66) of the isolates, respectively.

**Conclusions:**

There are two predominant lineages of *H. pylori* circulating in Vietnam; most of the isolates belong to the hspEastAsia population. The hpEurope population is further divided into two smaller clusters.

**Supplementary Information:**

The online version contains supplementary material available at 10.1186/s13099-021-00452-2.

## Introduction

*Helicobacter pylori* is an important human pathogen that is likely to be present in gastric mucosa of over half of the world’s population. The prevalence of *H. pylori* infection appears to be higher in the low- and middle- income countries than developed countries, with infection prevalence between ethnic groups within countries often varied [1, 2]. Such localised differences might be attributable to socioeconomic factors [4–6], although *H. pylori* related issues may contribute. The prevalence of infection in Asia and Africa is 54.7% to 79.1%, respectively. In North- and South- America the prevalence is 37.1% and 63.4%, respectively and in Europe, the prevalence is on averages 47.0% [3]. Prevalence differences between racial and ethnic groups have been described in various parts of the world, but the extent to which such differences can be attributed to socioeconomic and other possible risk factors is unclear [4–6]. Vietnam is the easternmost mainland country in Southeast Asia with an estimated population of 96 million (2019, UNFPA-VN) among which there are more than 50 ethnic groups of different cultures; ~ 65% of these groups are located exclusively in remote or rural areas (2019, UNFPA-VN) [7, 8]. Earlier studies in both hospital and community settings showed a high prevalence of *H.* *pylori* infection in Vietnam [9–11]. There is considerable variation in socioeconomic status and lifestyle across a rapidly changing Vietnam, this study investigates the risk associated with *H.* *pylori* infection in a major urban community in southern Vietnam building on previous studies [9–13]. Importantly, this study examines international context of the *H. pylori* present in Vietnam in relation to the major *H. pylori* populations.

*H. pylori* has undergone localized co-evolution with humans for more than 60,000 years [14]. The pattern of distribution of *H. pylori* populations have a strong association with human migration and are named after the geographic regions historically associated with particular human populations [15] [16]. The pattern of distribution *H. pylori* populations is indicative of the epidemiology of this organism, being exclusively associated with humans and very localized transmission, almost vertical. Importantly, the incidence and severity of gastric disease associated with *H. pylori* infection is observed with particular *H. pylori* genetic types in particular regions of the world. For instance, in East Asian countries such as Japan and Korea the incidence of gastric cancer is higher relative to European and North American countries [17].

The cytotoxin associated gene pathogenicity island (CagPAI) is one of the major virulence determinants of *H. pylori*. Several virulence genes in the CagPAI trigger abnormal cellular signals in the host. This abnormal cell signalling is likely to contribute to *H. pylori*-infection associated disease, including gastric cancer (GC). The *cagA* gene, present in the CagPAI, is known to be an important virulence factor and plays a key role in pathogenesis. The *cagA* gene is not present in all *H. pylori* strains, more than 90% of *H. pylori* isolates from East Asian countries carry *cagA*, compared to 50–70% of isolates from the Western countries [18, 19]. Although, studies of *H. pylori* isolates from East Asia showed individuals carrying *cagA* positive strains have an increased risk of peptic ulcer disease (PUD) and/or GC, compared to those from Western countries carrying *cagA* positive strains [20–22]. Functionally, the protein encoded by *cagA* activates several signal transduction pathways that bind and disrupt the function of epithelial junctions, leading to aberrations in the functioning of the tight junction, cell polarity and cell differentiation in the host [23].

The *H. pylori* vacuolating cytotoxin A, encoded by the *vacA* gene, is endocytosed by the host cells and causing changes including membrane channel formation resulting in cytochrome c release which initiates apoptosis and a pro-inflammatory response [24]. Particular allelic variants of *vacA* and *cagA* are associated with *H.* *pylori-*associated disease sequelae. Allelic types are associated with *H. pylori* populations and are probably host-specific adaptive changes [25]. The typing scheme used for *vacA* is based on the middle (m) and signal (s) region of the gene with two types defined for each region; alleles: m1 m2 and S1 and S2 respectively. In vitro experiments showed s1m1 strains induce cell vacuolation more frequently than s1m2 or s2m2, from which it was inferred that the s1m1 was more cytotoxic [26].

Vietnam has emerged as a country with the highest age-standardized incidence rate (ASR) of GC (16.3 cases/100,000 for both sexes) in Southeast Asia (GLOBOCAN 2012; http://globocan.iarc.fr). Previous studies have also reported the high prevalence of *H. pylori* infection in Vietnam and its association with peptic ulcer diseases, active gastritis, atrophy, and intestinal metaplasia [27]. As part of this prospective cross-sectional study, we have used isolate genome sequencing to enable the investigation of the *H. pylori* population types circulating in symptomatic Vietnamese patients. The genomic relationship between isolates and gene typing for the *cagA* and *vacA* genes (derived from the genome sequence for each isolate) provide key baseline information for identifying bacterial associated risk factors for *H. pylori*-associated disease in Vietnam and how these risk factors compare with *H. pylori*-associated disease in other parts of the world.

## Materials and methods

### Patient and specimen collection

We conducted a prospective cross-sectional study among patients attending at Gastroenterology Department of Gia Dinh Hospital, Ho Chi Minh City, Vietnam from August 2016 to February 2017. Instead of random selection, only patients with symptoms of upper gastrointestinal discomfort, heartburn, gastric or duodenal ulcer were eligible for enrolment. Candidate patients were informed about the study procedure and written informed consent was obtained for participation. Sociodemographic and clinical information was collected for each patient using a structured questionnaire at the time of clinical presentation. An endoscopic examination was performed by a trained clinician and two biopsy specimens (one from the gastric antrum and one from the corpus) were collected from each patient using well-washed and disinfected fibre optic endoscopes (model GIF XQ 30; Olympus, Japan). The biopsy specimens were transported to the laboratory in Stuart transport medium at 4 °C.

### Isolation of *H. pylori*

Biopsy samples were vortexed vigorously for 5 min and plated on Brain Heart Infusion (BHI) agar (Oxoid Ltd, Hampshire, United Kingdom) supplemented with 7.5% sheep blood, 0.4% Isovitalex, and *H. pylori* Dent supplement (Oxoid, United Kingdom). Plates were incubated at 37 °C in an atmosphere of 5% O_2_, 15% CO_2_, and 80% N2 for 3 to 7 days. *H. pylori* colonies were identified based on their typical morphology, characteristic appearance on Gram staining, a positive urease test, and subsequently confirmed by MALDI_TOF (Bruker, Germany). Isolates were stored at minus 80 °C in 0.5 ml of brain heart infusion (BHI) broth with 20% glycerol.

### Genomic DNA extraction and genome sequencing

Revived isolates were subcultured on selective BHI solid medium containing 7.5% sheep blood and 0.4% isovitalex under microaerophilic conditions (5% O_2_, 15% CO_2_, 80% N_2_) at 37 °C for 3–5 days [28]. Genomic DNA was prepared from confluent growth using a commercial DNA extraction kit (Qiagen DNA Mini kit, Germany). Genomic libraries were prepared using the Nextera DNA sample preparation kit (Illumina, San Diego, USA). Library sequencing was performed on the Illumina MiSeq instrument using the V3-600 cycle, paired-end kit (Illumina, CA. USA). Readsets for isolates sequenced as part of this study are available at National Center for Biotechnology (NCBI) under BioProject PRJNA689207 https://www.ncbi.nlm.nih.gov/bioproject/PRJNA689207

### Bacterial genome assembly and annotation

Sequences were analysed using the Nullarbor pipeline (https://github.com/tseemann/nullarbor). In brief, low-quality bases and adaptor contamination were trimmed off with Trimmomatic [29], readsets with at least 35 × read depth of coverage were retained for analysis. Isolate purity was evaluated with Kraken (v0.10.5) 5 [30]. SPAdes (v.3.9.0) [31] and Prokka (v.1.12) were used for de novo assembly and genome annotation, respectively. [32]. We used tRNAScan and RNAmmer to identify tRNA and rRNA in the draft genomes, respectively [33, 34]. The identification of phage related regions was carried out using the PHASTER tool [35].

### Phylogenetic analysis

Forty-two [42] reference *H. pylori* genome sequences representing selected *H. pylori* populations were downloaded from the NCBI, details are shown in Additional file 1: Table S1. Reads from the reference strains and the isolates in this study were aligned to the *H. pylori* strain 26695 (Accession: NC_000915) reference genome sequence using the Burrows-Wheeler Aligner MEM (v 0.7.15-r1140) algorithm [36] as implemented in Snippy; the core genome alignment was used to construct an SNP-based phylogenetic tree using FastTree [37]. SNPs were identified using Freebayes (v1.0.2) under a haploid model, with a minimum depth of coverage of 10× and allelic frequency of 0.9 required to confidently call an SNP [38]. The phylogenetic tree was visualized using MEGA-X [39].

### Core genome and pan-genome analysis

OrthoMCL was used to identify orthologous clusters using predicted protein sequences from each of the studied isolates (minimum threshold of 50 amino acids in length with identity and e-value parameters were at 70% and 0.00001 respectively) [40]. The identified clusters were aligned against the EggNOG database to predict a functional category. Clusters that contained proteins with more than one domain with distinct categories were assigned multiple categories. The functional categories were graphically represented using R (http://www.R-project.org). Proteins that could not be classified were assigned to category S (hypothetical). Graphical overviews of categorized strain-specific genes were produced using R.

### Identification of virulence-associated genes and cag pathogenicity island

*H. pylori* virulence genes were obtained from VFDB [41]. Genes were detected using Abricate (https://github.com/tseemann/abricate) with a minimum 80% sequence identity and 90% gene coverage [42]. Virulence gene distribution across isolates was visualised using Phandango (https://jameshadfield.github.io/phandango/#/). A visual overview of differences in gene content was obtained using Blast Ring Image Generator (BRIG) [43] with isolate genome sequences aligned against cagPAI of *H. pylori* strain 26695 (typical HpEurope) or strain F57 (typical hspEAsia).

### Statistical analysis

Data analysis was performed using Statistical Package for Social Science (SPSS) software (IBM SPSS Statistics 23, NY USA). Baseline descriptive statistics were summarized for the variables of interest. Comparisons between groups were performed using either the chi-squared or Fisher’s exact tests for categorical variables; *t*-tests and the Mann–Whitney U-test were used for continuous variables. A two-sided *P* value of > 0.05 was considered statistically significant.

### Ethics statement

The ethical review committee of the National University Ho Chi Minh City, Vietnam approved the study (Approval No: 702/DHQG-KHCN). Written informed consent was mandatory for patient enrolment in the study. For patients < 18 years, written informed consent was obtained from a parent or guardian.

## Results

### Patient population

One hundred sixty-one patients were enrolled in the study from August 2016 to February 2017. Among the patients, 44.7% (72/161) were male. The age (median; interquartile range (IQR)) was 39.4; 32–48 years. Among the patients, 51.6% (83/161) presented with epigastralgia, 31.7% (51/161) with abdominal fullness and 23.0% (37/161) with indigestion. In endoscopic examination, 95.7% of patients had stomach inflammation including 74.5% (120/161) congestion, 37.9% (16/161) erosion, 26.1% (42/161) oedema (Additional file 2: Table S2). Among the patients, 57.1% (92/161) had a primary infection (diagnosed with *H.* *pylori* infection for the first time) and 42.8% (69/161) had secondary infections (i.e. had a previous history of *H. pylori* infection). There was no difference in age, sex, gender, smoking, alcohol consumption, clinical symptoms and endoscopic findings between primary and secondary infection, although the number of symptoms was higher in secondary infection patients. Among the 161 positive biopsy samples diagnosed for *H.* *pylori*, 156 were tested positive by rapid urease test and five samples by *H. pylori* antigen test. Initially, *H. pylori* was cultured from 59% (95/161) patients, although only 87.4% (83/95) of these isolates could be revived and analysed.

### Genome characteristics

Summaries of the read data set and draft genome for each of the 83 *H. pylori* isolates are presented in Table 1. The read depth coverage in each of the isolate read sets ranged from 38–456×. The draft genome sequences comprised of between 16 and 83 coting’s. Overall, the average genome size was 1.6 Mb with 38.94% G + C content. For each isolate, the annotated genome sequence comprised between1451 and 1589 protein coding regions (CDS) with ~ 92% of the genome used for protein coding.

**Table 1:** Genome statistics of the whole-genome sequences of the 83 *H. pylori* isolates in this study

S. No	Isolate	Accession number	Read depth coverage	No. of contigs		Genome size (bp)	No. of CDS	Coding percentage	G + C percentage	Lineage	CAG PAI	Vac allele	EPIYA Motif	
1	GD13	SRR13341718	56	32		1610603	1504	94.2	38.5	hpEurope	Negative (−)	s2m2	–	
2	GD14	SRR13341717	44	33		1630906	1540	94.8	39.1	hspEastAsia	Positive ( +)	s1m1	ABD	
3	GD15	SRR13341706	210	44		1611095	1508	95.4	39.5	hspEastAsia	Positive ( +)	s1m1	ABD	
4	GD16	SRR13341695	50	52		1678689	1572	91	38.5	hspEastAsia	Positive ( +)	s1m1	ABD	
5	GD17	SRR13341684	107	38		1629934	1546	91.1	39.2	hspEastAsia	Positive ( +)	s1m1	ABD	
6	GD18	SRR13341673	53	49		1674278	1568	91	39.3	hspEastAsia	Positive ( +)	s1m1	ABD	
7	GD19	SRR13341662	119	36		1563624	1480	90.9	39.6	hspEastAsia	Positive ( +)	s1m2	ABCC	
8	GD20	SRR13341651	203	64		1696341	1589	95.6	38.9	hspEastAsia	Positive ( +)	s1m1	ABD	
9	GD21	SRR13341640	113	28		1603954	1517	91.1	38.9	hspEastAsia	Positive ( +)	s1m2	ABD	
10	GD22	SRR13341636	156	29		1567287	1501	94.5	39.6	hspEastAsia	Positive ( +)	s1m2	ABD	
11	GD23	SRR13341716	148	49		1641514	1534	93.4	39.5	hpEurope	Negative (−)	s1m2	–	
12	GD24	SRR13341715	148	33		1568554	1491	94.1	39.5	hspEastAsia	Positive ( +)	s1m1	ABD	
13	GD25	SRR13341714	51	31		1598180	1519	99.1	38.5	hspEastAsia	Positive ( +)	s1m1	ABD	
14	GD26	SRR13341713	143	33		1634715	1549	94.8	39.5	hspEastAsia	Positive ( +)	s1m2	ABD	
15	GD29	SRR13341712	216	36		1628946	1534	94.5	39.5	hspEastAsia	Positive ( +)	s1m2	ABD	
16	GD30	SRR13341711	120	42		1611416	1502	94.6	39	hspEastAsia	Positive ( +)	s1m2	ABD	
17	GD31	SRR13341710	100	41		1642696	1540	90.7	39.4	hpEurope	Negative (−)	s1m2	–	
18	GD32	SRR13341709	161	30		1597373	1514	93.3	39.5	hspEastAsia	Positive ( +)	s1m1	ABD	
19	GD33	SRR13341708	90	36		1674481	1557	92.7	38.7	hspEastAsia	Positive ( +)	s1m2	ABD	
20	GD34	SRR13341707	82	41		1682613	1578	95.5	38.5	hspEastAsia	Positive ( +)	s1m1	ABD	
21	GD35	SRR13341705	170	24		1553118	1459	93.1	39.5	hspEastAsia	Positive ( +)	s1m2	ABC	
22	GD36	SRR13341704	250	34		1683121	1572	94.2	39.3	hpEurope	Negative (−)	s1m2	–	
23	GD37	SRR13341703	109	20		1590694	1503	91.1	39.5	hspEastAsia	Positive ( +)	s1m1	ABD	
24	GD38	SRR13341702	264	36		1570557	1501	94.1	39.4	hspEastAsia	Positive ( +)	s1m2	ABD	
25	GD39	SRR13341701	190	34		1637712	1542	94.4	36.9	hspEastAsia	Positive ( +)	s1m1	ABD	
26	GD40	SRR13341700	119	36		1577762	1492	92.8	38.9	hspEastAsia	Positive ( +)	s1m2	ABD	
27	GD42	SRR13341699	413	24		1580561	1502	94.7	38.7	hspEastAsia	Positive ( +)	s1m1	ABD	
28	GD43	SRR13341698	413	24		1580561	1502	94.5	38.7	hspEastAsia	Positive ( +)	s1m1	ABD	
29	GD44	SRR13341697	170	22		1646259	1551	95.8	39	hspEastAsia	Positive ( +)	s1m2	ABD	
30	GD45	SRR13341696	199	38		1634413	1536	94.6	38.5	hspEastAsia	Positive ( +)	s1m2	ABD	
31	GD46	SRR13341694	291	39		1639952	1534	93.6	38.9	hpEurope	Negative (-)	s1m2	–	
32	GD47	SRR13341693	251	31		1653247	1569	96.2	38.7	hspEastAsia	Positive ( +)	s1m1	ABD	
33	GD48	SRR13341692	158	38		1637838	1539	93.4	38.8	hpEurope	Negative (−)	s1m2	–	
34	GD49	SRR13341691	183	23		1577856	1503	93.9	38.9	hspEastAsia	Positive ( +)	s1m1	ABD	
35	GD50	SRR13341690	108	39		1643348	1540	90.5	38.6	hpEurope	Negative (−)	s1m2	–	
36	GD51	SRR13341689	360	31		1604541	1509	95.3	39.4	hspEastAsia	Positive ( +)	s1m2	ABD	
37	GD52	SRR13341688	184	35		1537698	1471	92.9	39.1	hspEastAsia	Positive ( +)	s1m2	ABD	
38	GD53	SRR13341687	175	34		1573076	1480	94.5	39	hspEastAsia	Positive ( +)	s1m2	ABD	
39	GD55	SRR13341686	238	43		1638622	1533	93.6	38.6	hpEurope	Negative (-)	s1m2	–	
40	GD56	SRR13341685	180	33		1635691	1548	95.3	38.6	hspEastAsia	Positive ( +)	s1m2	ABD	
41	GD57	SRR13341683	240	33		1621906	1517	95.8	38.5	hspEastAsia	Positive ( +)	s1m2	ABD	
42	GD58	SRR13341682	308	35		1596620	1513	94.7	38.6	hspEastAsia	Positive ( +)	s1m2	ABD	
43	GD59	SRR13341681	262	48		1655979	1548	93.5	38.4	hpEurope	Negative (−)	s1m2	–	
44	GD60	SRR13341680	178	31		1557741	1464	93.6	38.8	hspEastAsia	Positive ( +)	s1m2	ABD	
45	GD61	SRR13341679	163	22		1600212	1512	94.9	38.7	hspEastAsia	Positive ( +)	s1m2	ABD	
46	GD62	SRR13341678	210	25		1554217	1451	93.2	39	hspEastAsia	Positive ( +)	s1m2	ABD	
47	GD63	SRR13341677	200	28		1574015	1503	94.2	38.3	hspEastAsia	Positive ( +)	s1m2	ABD	
48	GD64	SRR13341676	278	29		1628242	1525	95.8	38.7	hspEastAsia	Positive ( +)	s1m2	ABD	
49	GD65	SRR13341675	208	27		1652774	1568	95.4	37.5	hspEastAsia	Positive ( +)	s1m1	ABD	
50	GD66	SRR13341674	145	36		1593774	1501	94.5	38.7	hspEastAsia	Positive ( +)	s1m1	ABD	
51	GD67	SRR13341672	122	32		1593031	1501	91.7	38.7	hspEastAsia	Positive ( +)	s1m2	ABD	
52	GD68	SRR13341671	161	29		1593992	1485	94.2	38.8	hspEastAsia	Positive ( +)	s1m1	ABD	
53	GD69	SRR13341670	294	33		1562632	1474	93.9	38.6	hspEastAsia	Positive ( +)	s1m1	ABD	
54	GD70	SRR13341669	305	36		1622171	1540	94.6	39	hspEastAsia	Positive ( +)	s1m1	ABD	
55	GD71	SRR13341668	456	34		1554719	1470	93.3	38.8	hspEastAsia	Positive ( +)	s1m1	ABD	
56	GD72	SRR13341667	76	46		1593519	1501	93.3	38.9	hspEastAsia	Positive ( +)	s1m2	ABD	
57	GD73	SRR13341666	67	31		1612805	1538	90.5	38.8	hspEastAsia	Positive ( +)	s1m1	ABD	
58	GD74	SRR13341665	84	37		1624940	1525	94.9	38.8	hspEastAsia	Positive ( +)	s1m1	ABD	
59	GD75	SRR13341664	133	26		1608952	1530	94.6	39.3	hspEastAsia	Positive ( +)	s1m1	ABD	
60	GD76	SRR13341663	174	37		1642815	1541	93.4	39.3	hpEurope	Negative (−)	s1m2	–	
61	GD77	SRR13341661	149	45		1636257	1530	93.3	39.2	hpEurope	Negative (−)	s1m2	–	
62	GD79	SRR13341660	144	28		1563358	1484	95.4	39.6	hspEastAsia	Negative (−)	s1m2	–	
63	GD80	SRR13341659	136	41		1667621	1556	95.7	38.8	hpEurope	Positive ( +)	s1m1	ABD	
64	GD81	SRR13341658	131	51		1668954	1565	94	38.4	hpEurope	Negative (−)	s1m2	–	
65	GD82	SRR13341657	49	38		1620888	1530	91.1	38.6	hspEastAsia	Positive ( +)	s1m1	ABD	
66	GD83	SRR13341656	119	30		1547013	1459	93.8	39.6	hspEastAsia	Positive ( +)	s1m1	ABD	
67	GD84	SRR13341655	122	34		1642913	1544	93.3	39.2	hpEurope	Negative (−)	s1m2	–	
68	GD85	SRR13341654	85	31		1565822	1490	91.4	39.3	hspEastAsia	Positive ( +)	s1m1	ABD	
69	GD86	SRR13341653	95	53		1659927	1562	92	38.8	hspEastAsia	Positive ( +)	s1m2	ABD	
70	GD87	SRR13341652	113	37		1642282	1536	90.5	39.2	hpEurope	Negative (−)	s1m2	–	
71	GD88	SRR13341650	162	31		1553891	1473	93.1	39.6	hspEastAsia	Negative (−)	s1m2	–	
72	GD89	SRR13341649	132	35		1605386	1506	92	39.4	hspEastAsia	Positive ( +)	s1m2	ABD	
73	GD90	SRR13341648	128	47		1675801	1567	95.5	39.1	hspEastAsia	Positive ( +)	s1m1	ABD	
74	GD91	SRR13341647	97	16		1563179	1486	90.3	39.4	hspEastAsia	Positive ( +)	s1m2	ABD	
75	GD92	SRR13341646	111	46		1565490	1461	91.2	39.5	hspEastAsia	Positive ( +)	s1m2	ABCC	
76	GD93	SRR13341645	38	83		1639304	1518	93.7	38.6	hpEurope	Positive ( +)	s1m2	ABD	
77	GD94	SRR13341644	94	56		1637406	1534	94	37.6	hspEastAsia	Positive ( +)	s1m1	ABD	
78	GD95	SRR13341643	150	47		1674995	1560	94.3	39.1	hpEurope	Negative (−)	s1m2	–	
79	GD96	SRR13341642	123	32		1553728	1478	89.9	39.4	hspEastAsia	Positive ( +)	s1m1	ABD	
80	GD97	SRR13341641	187	33		1571349	1473	94.3	39.6	hspEastAsia	Positive ( +)	s1m2	ABD	
81	GD98	SRR13341639	132	34		1642357	1552	95	39.1	hspEastAsia	Positive ( +)	s1m2	ABD	
82	GD99	SRR13341638	131	41		1641954	1552	94.8	38.4	hspEastAsia	Positive ( +)	s1m1	ABD	
83	GD100	SRR13341637	67	34		1673344	1574	91.1	38.3	hspEastAsia	Positive ( +)	s1m1	ABD	

Single and incomplete phage associated region (8.1–13.5 kb) was detected in 17% (14/83) of the draft genome sequences. The phage sequences consist of between nine and 14 CDSs that encode either putative restriction-modification protein, TMP kinase, PcrA helicase, putative transposase, or other hypothetical proteins in addition to phage related genes (Additional file 3: Table S3).

### Core and pan-genome analysis

**Fig. 1:**
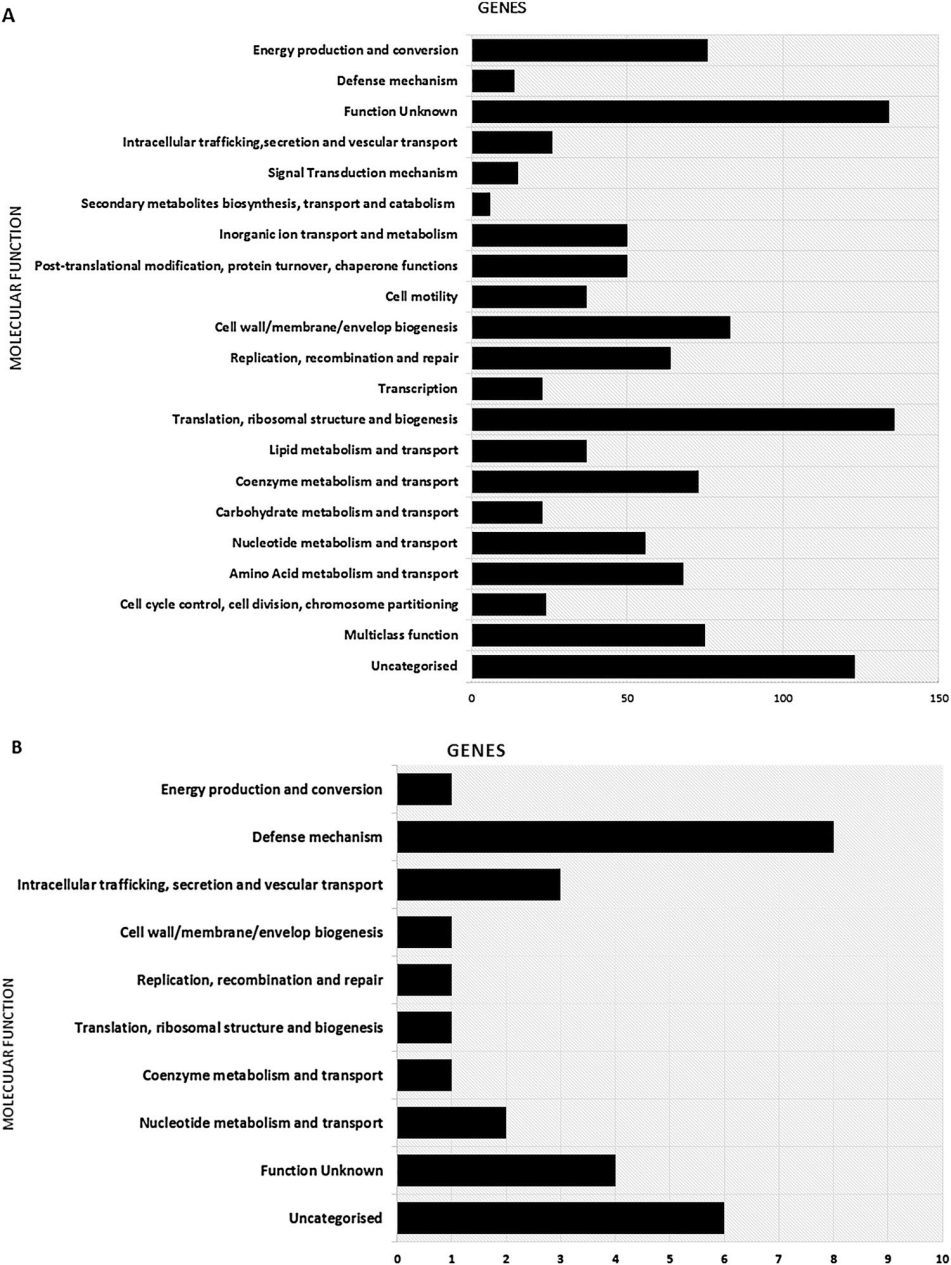
**A** Functional classification of 1,194 core orthologous clusters produced from the set of predicted proteins encoded on the genome sequence of each the 83 *H. pylori* study isolates using OrthoMCL. **B** Functional classification of the 28 isolate specific genes identified as part of the comparison of the protein coding capacity of the 83 study isolates. On the X-axis is the number of genes in each functional class on the Y-axis

The core- and pan- genome analysis by OrthoMCL identified 1,194 orthologous clusters (core genome) from the 119,366 annotated proteins in the 83 isolates. Among these 1070 orthologous clusters (core genome) were assigned functional categories using EggNOG database (Fig. 1a). A high proportion (12.7%, 136/1,070 and 7.7%, 83/1070) of the classified clusters belonged to the J (translation, ribosomal structure, and biogenesis), and M (cell membrane/envelope biogenesis) functional category, respectively. Proteins with no orthologues were detected in a small number of isolates, 26% (31/83) isolates contained either one or two proteins of this type. Most of these unique proteins were V (defence mechanism) or S (hypothetical) functional categories (Fig. 1b).

### Phylogenetic analysis

**Fig. 2:**
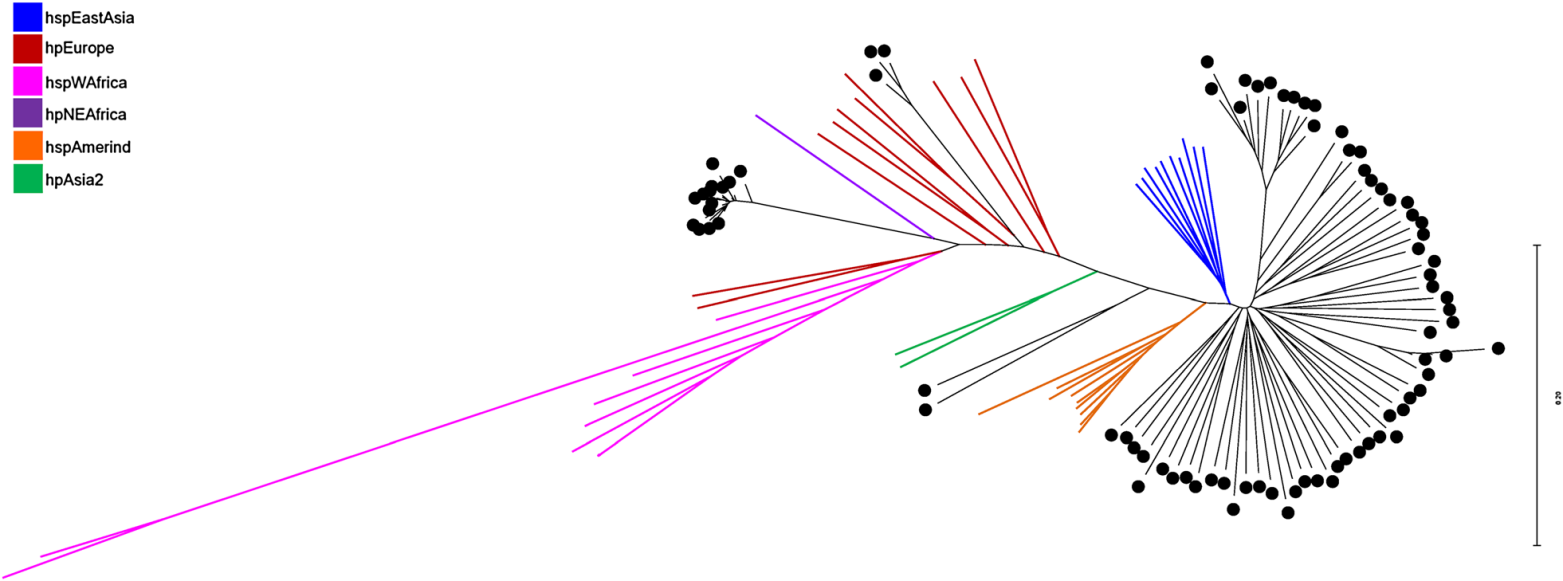
A tree showing the core genome relationship between the 83 Vietnamese *H. pylori* isolates and 42 *H. pylori* reference genomes. The 83 Vietnamese isolates are indicated by black terminal branches, while classified isolates are shown with coloured terminal branches as follows: hspEastAsia (blue), hpEurope (brown), hspWAfrica (pink), hpNEAfrica (purple), hspAmerind (orange) and hpAsia2 (green). The tree was inferred using the core genome comparison method as implemented in Nullarbor with *H. pylori* strain 26695 (Accession: NC_000915) used as the reference genome sequence for read mapping. The tree was modified using tools available in FigTree and MEGA-X

The genomic relationship between the 83 study isolates and 42 reference genome sequences for which the *H. pylori* population type was known was inferred from the core genome using the *H. pylori* strain 26,695 (Accession: NC_000915) as the reference genome sequence for read mapping. The tree shown in Fig. 2 provides a visual summary of the relationship between isolates. The core genome comparison showed that 80% (66/83) of the isolates were part of the *H. pylori* hspEastAsia population and the remaining 20%, 17/83 of isolates were part of the *H. pylori* hpEurope population based on the core genome relationship with the 42 classified isolates (Fig. 2).

### Virulence factors

**Fig. 3:**
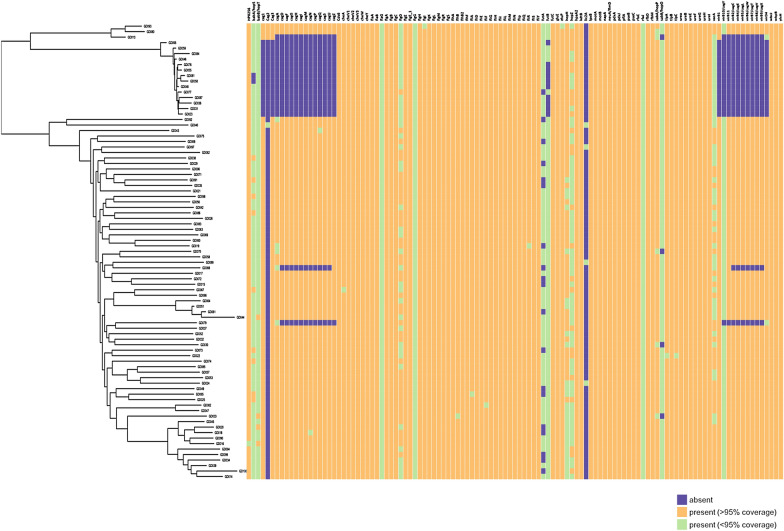
At the left there is a tree showing the core genome relationship between the 83 Vietnamese isolates. The virulence gene content for each of the isolates is colour coded at the right. Virulence genes detected are those present in the VFDB, virulence genes were detected using Abricate. (Green shows genes detected with less than 90% gene coverage, while Orange shows genes detected with greater than 90% gene coverage. Purple shows the gene was not detected

Virulence factors detection using the VFDB showed that 80% (66/83) Vietnamese isolates harboured between 110 and 113 virulence genes including all CagPAI genes and the *vacA* virulence genes whereas, 20% (17/83) of isolates contained 83 to 92 virulence genes. The second group of isolates usually lacked the *cag1* to *cag3* and *cagA* to *cagZ* genes of the CagPAI. Genes encoding urease enzymes, most of the flagella associated proteins, some endotoxins, and most of the Lewis antigens such as FutB, FutC and NeuA/FlmD were detected in all isolates (Fig. 3).

**Fig. 4:**
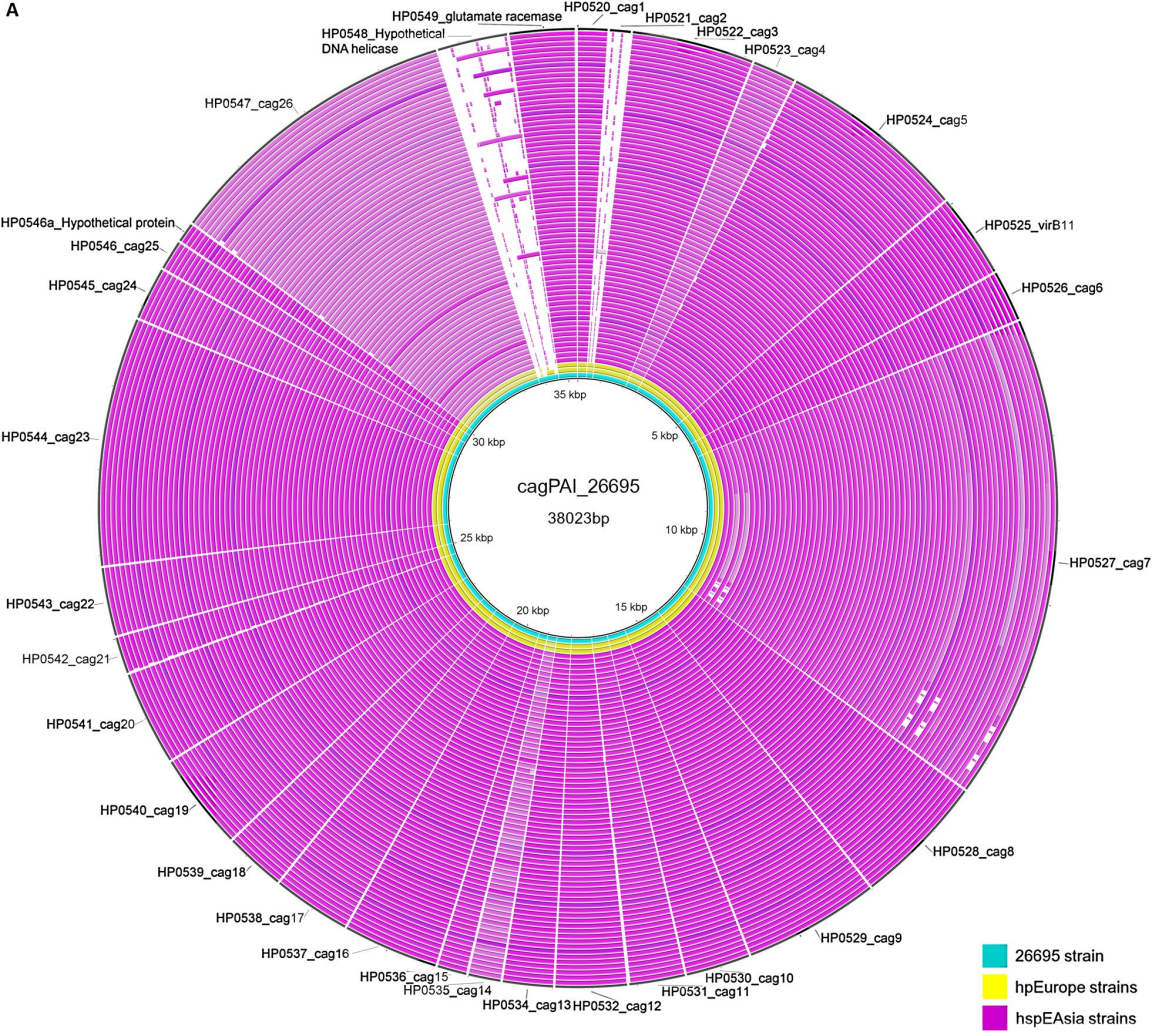
Comparison of the genetic organization of cagPAI of Vietnamese *H. pylori* isolates with a Western-type cagPAI (*H. pylori* strain 26695). The innermost blue ring shows the strain 26695 sequence with the HpEurope classified Vietnamese isolates shown as yellow rings and hspEastAsia Vietnamese isolates shown as pink rings. The Figure was constructed using BRIG

The virulence properties of the isolates are presented in Table 2. A complete CagPAI was present in 80% (66/83) of the genomes; of these, 97% (64/66) CagPAI positive isolates belonged to hspEastAsia population and the remaining 3% (2/66) belonged to hpEurope population (Table 2).Among 17 hpEurope isolates, 15 were CagPAI negative. Most of the CagPAI positive hspEastAsia and hpEurope isolates lacked an orthologue to the DNA helicase (HP0548) present in the Western-type CagPAI sequence found in *H. pylori* strain 26695 (Fig. 4).

**Table 2:** *H. pylori* virulence factors (*cagA* and *vacA*) in study isolates

Genotypes description	Total n = 83 n (%)	Lineage classification	
		hspEastAsia n = 66	hpEurope n = 17
*cagA genotype*			
*cagA-positive*	66 (79.5%)	64/66	2/66
ABD	63 (75.9%)	61/63	2/63
ABC	1 (1.2%)	1/1	0/1
ABCC	2 (2.4%)	2/2	0/2
Pre-EPIYA type -no deletion	34 (41%)	32/34	2/34
Pre-EPIYA type -18 bp deletion	31 (37.3%)	30/31	1/31
Pre-EPIYA type -39 bp deletion	1 (1.2%)	1/1	0/1
*cagA negative*	17 (20.5%)	2/17	15/17
*vacA*	83 (100%)	66/66	17/17
s1	82 (98.8%)	66/66	16/17
s2	1 (1.2%)	0/66	1/17
m1	34 (41%)	33/66	1/17
m2	49 (59%)	33/66	16/17
Genotype summary			
*cagA*-positive/*vacA* s1m1	34	33	1
*cagA*-negative/*vacA* s1m1	0	0	0
*cagA*-positive/*vacA* s1m2	32	31	1
*cagA*-negative/*vacA* s1m2	16	2	14
*cagA*-positive/*vacA* s2m2	0	0	0
*cagA*-negative/*vacA* s2m2	1	0	1
Total	83	66	17

Sequence analyses of the second repeat region of the *cagA* gene revealed that 95% (63/66), including two hpEurope isolates were of the ABD type, while the remaining three isolates (all hspEastAsia) were EPIYA-ABC or EPIYA-ABCC types (Table 2). Two hpEurope isolates had ABD type second repeat region of the *cagA* gene, which is an atypical characteristic of hpEurope strains. We also found 5% (3/63) of isolates containing an East Asian type *cagA* contained EPIYA-like sequences, ESIYA at EPIYA-B segments. Three vacA types were detected among the Vietnamese isolates, 34 isolates were s1m1 type, 48 isolates were s1m2 type and one isolate was s2m2 type. The most frequent genotypes among the *cagA* positive isolates were *vacA* s1m1/*cagA* + and *vacA* s1m2/ *cagA* + , accounted 51.5% (34/66) and 48.5% (32/66) of isolates, respectively.

## Discussion

*H. pylori* infection is associated with the development of gastric disease in the host; the frequency of infection and frequency of disease in the host varies across the world but there is an association between particular *H. pylori* genetic types in particular geographic regions with the disease. Developing effective strategies to manage *H. pylori*-associated disease relies on understanding the local *H. pylori* populations. This in conjunction with the significant *H. pylori*-associated disease burden in Vietnam highlights the important knowledge gap addressed by this study. Herein, we present genomic and epidemiological data for 83 Vietnamese *H. pylori* isolates. The frequency of *H. pylori* isolation was 59% (95/161) from the biopsies of symptomatic patients. This is similar to the result of earlier studies, where 270 randomly selected patients who underwent esophagogastroduodenoscopy at the endoscopy centres at either of two major hospitals in Hanoi and Ho Chi Minh (the biggest city in Northern and Southern Vietnam, respectively) [27]. Our phylogenetic data show that most *H. pylori* isolates from symptomatic Vietnamese patients are from the hspEastAsia population (80% of isolates). The dominance of the hspEastAsia population is consistent with the *H. pylori* population being strongly associated with human migration [16] where historical and emigrational evidence suggests the Vietnamese are more related to people from North Asia than to people from South Asia [44]. Moreover, migratory patterns with North Asia would have been influenced by the fact that Vietnam was under Chinese occupation for over a thousand years. Notably, a group of the Vietnamese isolates form an exclusive clade within the hspEAsia population, perhaps indicating that the Vietnamese were isolated from other South East Asian populations for an extended period; this may be supported by a study by Breurec et al. showing Khmer and Vietnamese isolates as deep branching members of the hspEastAsia *H. pylori* population [45]. More extensive sampling of *H. pylori* in the region would be required to confirm a *H. pylori* subpopulation for Vietnam. The Vietnamese *H.* *pylori* isolates that are part of the hpEurope population are likely to have arisen through the French colonial occupation of Vietnam and other parts of South East Asia during the 19th and early 20th centuries. We observe a small number of isolates that appear to be related to the representative isolates from the hpNEAfrica or hspWAfrica population used in our comparative analysis (Fig. 2). Another possibility is that these isolates are recombinant hybrids arising from the endemic hspEastAsia and hpEurope population strains now present in Vietnam [45].

The prevalence of *H. pylori* infection has been reported in between 50 to 80% in several studies conducted in adults in Vietnam, this is similar to Japan, Korea or China, and other South Asian nations [9–11, 46, 47]. The genetic characteristics and diversity of Vietnamese *H. pylori* strains could be a factor contributing to the high incidence of gastric cancer in Vietnam. Evidence indicates that the isoforms of *vacA* and the type and number of the EPIYA motifs in the *cagA* gene strongly influence the type and magnitude of the histological damage of the gastric mucosa. For example, the *vacA* s1m1 genotype has been associated with intestinal metaplasia, severe inflammation and a high risk of gastric cancer [20, 48, 49]. In this study, the s1m1*vacA* allelic combination was detected in 41% of isolates. In addition, East Asian *cagA*, which is more prevalent in Vietnamese isolates is more frequently associated with disease than Western *cagA* [20, 50, 51]. This study revealed a lower frequency of *cagA* than previous reports on Vietnamese *H. pylori* [52–55] which may contribute to the lower rates of gastric ulcer and gastric cancer observed in Vietnam. In dyspeptic patients from central Vietnam, the frequency of *cagA* + strains was 84% [54]. In *H. pylori* strains from Southern Vietnam with gastric cancer and peptic ulcer, all strains were *cagA* positive [52]. In this study, the *cagA* was found frequently with the *vacA* s1m1 allelic type (51.5%, 34/66), which is consistent with previous reports from South or North Vietnam isolates [27, 55]. The most frequent EPIYA motif found in our isolates was ABD (96.6%; 63/66), which is similar to previous reports from Vietnamese patients with the gastric disease [52, 55]. However, these frequencies were different in central Vietnam isolates, where *vacA* s1m1/ *cagA* + genotype was detected in 64.86% (48/74) of isolates and the *cagA*–ABD motif was found in a lower proportion (91%) [54].

We observed that 88.2% (15/17) of hpEurope isolates were either negative or possibly lost their *cagA* during the course of evolution or, if present, they had ABD type EPIYA-motif. The presence of ABD type EPIYA-motif pattern is an atypical characteristic of hpEurope strains where ABC type EPIYA-motif is more prevalent. The gene content and organization of genes of cagPAI are highly conserved. The phylogeny of most cagPAI genes including *cagA* was found to be similar to that of housekeeping genes, indicating that the cagPAI was probably acquired only once by *H. pylori* [56]. Recombination events during mixed infection have been identified as a major driving force behind allelic diversity in *H. pylori* cagPAI largely reflects that of *H. pylori*’s housekeeping genes being under diversifying selection or positive selection due to host polymorphisms which could even result in modified host protein interactions [56]. Accordingly, hpEurope and hspEastAsia strains are expected to carry a Western and an East Asian *cagA* respectively. A prominent example of amino acid diversity noted previously are the EPIYA motifs in the C- terminal half of *cagA*, which differ between Asian (hpAsia2; hspEastAsia) (type D) and all other populations [57]. The D type EPIYA repeat binds SHP-2 phosphatase more avidly than other types [22]. Furthermore, Furuta Y. et al. also clarified the recombination-mediated routes of *cagA* evolution and provided a solid basis for a deeper understanding of its function in pathogenesis [58]. Based on this observation, the predominant host may be applying a selective pressure on Vietnamese hpEurope strains for the ABD type *cagA* that is normally observed in the *cagA* of hspEastAsia lineage strains.

## Conclusions

Our study confirmed the high prevalence of *H. pylori* infection and the most virulent genotypes combination *vacA* s1m1/*cagA* + in *H. pylori* isolates recovered from Vietnamese symptomatic patients, which may explain the higher incidence rate of gastric cancer in Vietnam. Our data on the genetic architecture of *H. pylori* strains isolated from symptomatic Vietnamese patients showed two predominant lineages, with the majority of isolates belonging to the hspEastAsia population. However, there is another group of Vietnamese isolates that is part of hpEurope population. Interestingly, the hpEurope population isolates are divided into two subclusters. Although phylogeny has been improved by increasing the number of genes analyzed, analyses of a limited number of genes cannot uncover more complex evolutionary events. Our study also has a limitation that almost all our enrolled patients were in the early stage of gastric diseases, so we could not explore the interaction between *H. pylori* genotypes and their outcomes.

## Supplementary Information

**Additional file 1:Table S1.** H. pylori reference strains used in this study.

**Additional file 2:Table S2.** Sociodemographic, behavioral, clinical information of the 161 patients^ included in the study.

**Additional file 3:Table S3.** Putative phage regions identified in isolates using PHASTER tool and major genes encoded within these regions.

## Data Availability

All sequence data are available at National Centre for Biotechnology (NCBI) under BioProject PRJNA689207 (https://dataview.ncbi.nlm.nih.gov/object/PRJNA689207?reviewer=tjt9hpdp22vgbolmppp4r2fc5s). All other data and materials used for this publication are available under the OUCRU data sharing policy and can be requested at DAC@oucru.org.

## Abbreviations

ASR: Age-standardized incidence rate
BHI: Brain heart infusion
CagPAI: Cytotoxin associated gene pathogenicity island
CDS: Coding sequences
GC: Gastric cancer
NCBI: National Center for Biotechnology
PUD: Peptic ulcer disease
SNP: Single Nucleotide Polymorphism
SPSS: Statistical Package for Social Science
VacA: Vacuolating cytotoxin A
UNFPA-VN: United Nations Population Fund-Vietnam
VFDB: Virulence Factor Database

## References

[1] Fox JG, Yan LL, Dewhirst FE, Paster BJ, Shames B, Murphy JC, Hayward A, Belcher JC, Mendes EN (1995). Helicobacter bilis sp. nov., a novel helicobacter species isolated from bile, livers, and intestines of aged, inbred mice. J Clin Microbiol.

[2] Eusebi LH, Zagari RM, Bazzoli F (2014). Epidemiology of *Helicobacter**pylori* infection. Helicobacter.

[3] Hooi JKY, Lai WY, Ng WK, Suen MMY, Underwood FE, Tanyingoh D, Malfertheiner P, Graham DY, Wong VWS, Wu JCY (2017). Global prevalence of helicobacter pylori infection: systematic review and meta-analysis. Gastroenterology.

[4] Malaty HM, El-Kasabany A, Graham DY, Miller CC, Reddy SG, Srinivasan SR, Yamaoka Y, Berenson GS (2002). Age at acquisition of *Helicobacter**pylori* infection: a follow-up study from infancy to adulthood. Lancet.

[5] Kivi M, Johansson AL, Reilly M, Tindberg Y (2005). Helicobacter pylori status in family members as risk factors for infection in children. Epidemiol Infect.

[6] Epplein M, Signorello LB, Zheng W, Peek RM, Michel A, Williams SM, Pawlita M, Correa P, Cai Q, Blot WJ (2011). Race, African ancestry, and *Helicobacter**pylori* infection in a low-income United States population. Cancer Epidemiol Biomarkers Prev.

[7] Rheinlander T, Samuelsen H, Dalsgaard A, Konradsen F (2010). Hygiene and sanitation among ethnic minorities in Northern Vietnam: does government promotion match community priorities?. Soc Sci Med.

[8] Vietnam. Ban chỉ đạo Tỏ̂ng điè̂u tra dân só̂ và nhà ở trung ương (2010). The 2009 Vietnam population and housing census.

[9] Hoang TT, Bengtsson C, Phung DC, Sorberg M, Granstrom M (2005). Seroprevalence of *Helicobacter**pylori* infection in urban and rural Vietnam. Clin Diagn Lab Immunol.

[10] Nguyen VB, Nguyen GK, Phung DC, Okrainec K, Raymond J, Dupond C, Kremp O, Kalach N, Vidal-Trecan G (2006). Intra-familial transmission of *Helicobacter**pylori* infection in children of households with multiple generations in Vietnam. Eur J Epidemiol.

[11] Nguyen BV, Nguyen KG, Phung CD, Kremp O, Kalach N, Dupont C, Raymond J, Vidal-Trecan G (2006). Prevalence of and factors associated with *Helicobacter**pylori* infection in children in the north of Vietnam. Am J Trop Med Hyg.

[12] Nguyen TV, Nguyen VB (2017). Prevalence and risk factors of *Helicobacter**pylori* infection in Muong children in Vietnam. Ann Clin Lab Res.

[13] Nguyen LX (2007). Epidemiological features of *Helicobacter**pylori* infection in children of five different ethnics in mountainous village. J Med Res.

[14] Linz B, Balloux F, Moodley Y, Manica A, Liu H, Roumagnac P, Falush D, Stamer C, Prugnolle F, van der Merwe SW (2007). An African origin for the intimate association between humans and *Helicobacter**pylori*. Nature.

[15] Yamaoka Y (2009). *Helicobacter**pylori* typing as a tool for tracking human migration. Clin Microbiol Infect.

[16] Falush D, Wirth T, Linz B, Pritchard JK, Stephens M, Kidd M, Blaser MJ, Graham DY, Vacher S, Perez-Perez GI (2003). Traces of human migrations in *Helicobacter**pylori* populations. Science.

[17] Bickenbach K, Strong VE (2012). Comparisons of gastric cancer treatments: East vs West. J Gastric Cancer.

[18] Yamaoka Y, Kodama T, Kashima K, Graham DY, Sepulveda AR (1998). Variants of the 3′ region of the cagA gene in *Helicobacter**pylori* isolates from patients with different H. pylori-associated diseases. J Clin Microbiol.

[19] Yamaoka Y, Orito E, Mizokami M, Gutierrez O, Saitou N, Kodama T, Osato MS, Kim JG, Ramirez FC, Mahachai V (2002). *Helicobacter**pylori* in North and South America before Columbus. FEBS Lett.

[20] Jones KR, Joo YM, Jang S, Yoo YJ, Lee HS, Chung IS, Olsen CH, Whitmire JM, Merrell DS, Cha JH (2009). Polymorphism in the CagA EPIYA motif impacts development of gastric cancer. J Clin Microbiol.

[21] Vilaichone RK, Mahachai V, Tumwasorn S, Wu JY, Graham DY, Yamaoka Y (2004). Molecular epidemiology and outcome of *Helicobacter**pylori* infection in Thailand: a cultural cross roads. Helicobacter.

[22] Azuma T, Yamakawa A, Yamazaki S, Ohtani M, Ito Y, Muramatsu A, Suto H, Yamazaki Y, Keida Y, Higashi H (2004). Distinct diversity of the cag pathogenicity island among *Helicobacter**pylori* strains in Japan. J Clin Microbiol.

[23] Backert S, Tegtmeyer N, Selbach M (2010). The versatility of *Helicobacter**pylori* CagA effector protein functions: the master key hypothesis. Helicobacter.

[24] Roesler BM, Rabelo-Goncalves EM, Zeitune JM (2014). Virulence factors of *Helicobacter**pylori*: a review. Clin Med Insights Gastroenterol.

[25] Kusters JG, van Vliet AH, Kuipers EJ (2006). Pathogenesis of *Helicobacter**pylori* infection. Clin Microbiol Rev.

[26] Yamaoka Y (2010). Mechanisms of disease: *Helicobacter**pylori* virulence factors. Nat Rev Gastroenterol Hepatol.

[27] Nguyen TL, Uchida T, Tsukamoto Y, Trinh DT, Ta L, Mai BH, Le SH, Thai KD, Ho DD, Hoang HH (2010). *Helicobacter**pylori* infection and gastroduodenal diseases in Vietnam: a cross-sectional, hospital-based study. BMC Gastroenterol.

[28] Nahar S, Mukhopadhyay AK, Khan R, Ahmad MM, Datta S, Chattopadhyay S, Dhar SC, Sarker SA, Engstrand L, Berg DE (2004). Antimicrobial susceptibility of *Helicobacter**pylori* strains isolated in Bangladesh. J Clin Microbiol.

[29] Bolger AM, Lohse M, Usadel B (2014). Trimmomatic: a flexible trimmer for Illumina sequence data. Bioinformatics.

[30] Wood DE, Salzberg SL (2014). Kraken: ultrafast metagenomic sequence classification using exact alignments. Genome Biol.

[31] Bankevich A, Nurk S, Antipov D, Gurevich AA, Dvorkin M, Kulikov AS, Lesin VM, Nikolenko SI, Pham S, Prjibelski AD (2012). SPAdes: a new genome assembly algorithm and its applications to single-cell sequencing. J Comput Biol.

[32] Seemann T (2014). Prokka: rapid prokaryotic genome annotation. Bioinformatics.

[33] Lowe TM, Eddy SR (1997). tRNAscan-SE: a program for improved detection of transfer RNA genes in genomic sequence. Nucleic Acids Res.

[34] Lagesen K, Hallin P, Rodland EA, Staerfeldt HH, Rognes T, Ussery DW (2007). RNAmmer: consistent and rapid annotation of ribosomal RNA genes. Nucleic Acids Res.

[35] Arndt D, Grant JR, Marcu A, Sajed T, Pon A, Liang Y, Wishart DS (2016). PHASTER: a better, faster version of the PHAST phage search tool. Nucleic Acids Res.

[36] Li H, Durbin R (2009). Fast and accurate short read alignment with Burrows-Wheeler transform. Bioinformatics.

[37] Price MN, Dehal PS, Arkin AP (2010). FastTree 2–approximately maximum-likelihood trees for large alignments. PLoS ONE.

[38] Garrison EMG. Haplotype-based variant detection from short-read sequencing. arXiv. preprint arXiv 2012. https://arxiv.org/abs/1207.3907

[39] Kumar S, Stecher G, Li M, Knyaz C, Tamura K (2018). MEGA X: molecular evolutionary genetics analysis across computing platforms. Mol Biol Evol.

[40] Li L, Stoeckert CJ, Roos DS (2003). OrthoMCL: identification of ortholog groups for eukaryotic genomes. Genome Res.

[41] Chen L, Yang J, Yu J, Yao Z, Sun L, Shen Y, Jin Q (2005). VFDB: a reference database for bacterial virulence factors. Nucleic Acids Res.

[42] Qumar S, Nguyen TH, Nahar S, Sarker N, Baker S, Bulach D, Ahmed N, Rahman M (2020). A comparative whole genome analysis of *Helicobacter**pylori* from a human dense South Asian setting. Helicobacter.

[43] Alikhan NF, Petty NK, Ben Zakour NL, Beatson SA (2011). BLAST Ring Image Generator (BRIG): simple prokaryote genome comparisons. BMC Genomics.

[44] Pischedda S, Barral-Arca R, Gomez-Carballa A, Pardo-Seco J, Catelli ML, Alvarez-Iglesias V, Cardenas JM, Nguyen ND, Ha HH, Le AT (2017). Phylogeographic and genome-wide investigations of Vietnam ethnic groups reveal signatures of complex historical demographic movements. Sci Rep.

[45] Breurec S, Guillard B, Hem S, Brisse S, Dieye FB, Huerre M, Oung C, Raymond J, Tan TS, Thiberge JM (2011). Evolutionary history of *Helicobacter**pylori* sequences reflect past human migrations in Southeast Asia. PLoS ONE.

[46] Quach DT, Vilaichone RK, Vu KV, Yamaoka Y, Sugano K, Mahachai V (2018). *Helicobacter**pylori* infection and related gastrointestinal diseases in Southeast Asian Countries: an expert opinion survey. Asian Pac J Cancer Prev.

[47] Asaka M, Kimura T, Kudo M, Takeda H, Mitani S, Miyazaki T, Miki K, Graham DY (1992). Relationship of Helicobacter pylori to serum pepsinogens in an asymptomatic Japanese population. Gastroenterology.

[48] Zhou W, Yamazaki S, Yamakawa A, Ohtani M, Ito Y, Keida Y, Higashi H, Hatakeyama M, Si J, Azuma T (2004). The diversity of vacA and cagA genes of *Helicobacter**pylori* in East Asia. FEMS Immunol Med Microbiol.

[49] Sahara S, Sugimoto M, Vilaichone RK, Mahachai V, Miyajima H, Furuta T, Yamaoka Y (2012). Role of *Helicobacter**pylori* cagA EPIYA motif and vacA genotypes for the development of gastrointestinal diseases in Southeast Asian countries: a meta-analysis. BMC Infect Dis.

[50] Singh K, Ghoshal UC (2006). Causal role of Helicobacter pylori infection in gastric cancer: an Asian enigma. World J Gastroenterol.

[51] Chattopadhyay S, Patra R, Chatterjee R, De R, Alam J, Ramamurthy T, Chowdhury A, Nair GB, Berg DE, Mukhopadhyay AK (2012). Distinct repeat motifs at the C-terminal region of CagA of *Helicobacter**pylori* strains isolated from diseased patients and asymptomatic individuals in West Bengal, India. Gut Pathog.

[52] Truong BX, Mai VT, Tanaka H, le Ly T, Thong TM, Hai HH, Van Long D, Furumatsu K, Yoshida M, Kutsumi H (2009). Diverse characteristics of the CagA gene of *Helicobacter**pylori* strains collected from patients from southern vietnam with gastric cancer and peptic ulcer. J Clin Microbiol.

[53] Nguyen LT, Uchida T, Tsukamoto Y, Trinh TD, Ta L, Mai HB, Le HS, Ho DQ, Hoang HH, Matsuhisa T (2010). Clinical relevance of cagPAI intactness in *Helicobacter**pylori* isolates from Vietnam. Eur J Clin Microbial Infect Dis.

[54] Phan TN, Santona A, Tran VH, Tran TNH, Le VA, Cappuccinelli P, Rubino S, Paglietti B (2017). Genotyping of *Helicobacter**pylori* shows high diversity of strains circulating in central Vietnam. Infect Genet Evol.

[55] Uchida T, Nguyen LT, Takayama A, Okimoto T, Kodama M, Murakami K, Matsuhisa T, Trinh TD, Ta L, Ho DQ (2009). Analysis of virulence factors of *Helicobacter**pylori* isolated from a Vietnamese population. BMC Microbiol.

[56] Olbermann P, Josenhans C, Moodley Y, Uhr M, Stamer C, Vauterin M, Suerbaum S, Achtman M, Linz B (2010). A global overview of the genetic and functional diversity in the *Helicobacter**pylori* cag pathogenicity island. PLoS Genet.

[57] Suerbaum S, Josenhans C (2007). Helicobacter pylori evolution and phenotypic diversification in a changing host. Nat Rev Microbiol.

[58] Furuta Y, Yahara K, Hatakeyama M, Kobayashi I (2011). Evolution of cagA oncogene of *Helicobacter**pylori* through recombination. PLoS ONE.

