# Supplementary material for: The endemic Helicobacter pylori population in Southern Vietnam has both South East Asian and European origins
Source: Gut Pathog. 2021 Sep 30;13:57. doi: 10.1186/s13099-021-00452-2 (PMC8482589; doi:10.1186/s13099-021-00452-2)
Supplement: Supplementary file 1 — Additional file 1:Table S1. H. pylori reference strains used in this study. [file 13099_2021_452_MOESM1_ESM.docx]

**S Table 1**: *H. pylori* reference strains used in this study

| **No.** | **NCBI** | **Strain** | **Lineage** | **Country** | **Remark** |  |
| --- | --- | --- | --- | --- | --- | --- |
| 1 | CP002331 | India7 | hpAsia2 | India |  |  |
| 2 | CP002983 | SNT49 | hpAsia2 | India |  |  |
| 3 | NC_000915 | 26695 | hpEurope | UK |  |  |
| 4 | CP002953 | ELS37 | hpEurope | El Salvado |  |  |
| 5 | NC_011333.1 | G27 | hpEurope | Italia |  |  |
| 6 | CP007603 | J166 | hpEurope | USA |  |  |
| 7 | NC_017362 | Lithuania75 | hpEurope | Lithuania |  |  |
| 8 | CP001217 | P12 | hpEurope | Europe |  |  |
| 9 | CP002073 | sjm180 | hpEurope | Peru |  |  |
| 10 | NC_008086 | HPAG1 | hpEurope | Sweden |  |  |
| 11 | NC_014256 | B8 | hpEurope | USA |  |  |
| 12 | NC_012973 | B38 | hpEurope | Europe |  |  |
| 13 | CP002076 | Cuz20 | hspAmerind | Peru |  |  |
| 14 | CP002980 | Puno120 | hspAmerind | Peru |  |  |
| 15 | NC_017359 | Sat464 | hspAmerind | Peru |  |  |
| 16 | NC_010698 | Shi470 | hspAmerind | Peru |  |  |
| 17 | CP003472 | Shi417 | hspAmerind | Peru |  |  |
| 18 | NC_017741 | Shi112 | hspAmerind | Peru |  |  |
| 19 | NC_017355 | v225d | hspAmerind | Venezuela |  |  |
| 20 | CP002074 | PeCan4 | hspAmerind | Peru |  |  |
| 21 | NC_017379 | Puno135 | hspAmerind | Peru |  |  |
| 22 | NZ_CP011483 | DU15 | hspEAsia | Korea |  |  |
| 23 | AP011943.1 | F32 | hspEAsia | Japan |  |  |
| 24 | NC_017367 | F57 | hspEAsia | Japan |  |  |
| 25 | CP003419 | XZ274 | hspEAsia | China |  |  |
| 26 | NC_017382 | 51 | hspEAsia | Korea |  |  |
| 27 | NC_017354 | 52 | hspEAsia | Korea |  |  |
| 28 | NC_017360 | 35A | hspEAsia | Japan |  |  |
| 29 | NC_017368 | F16 | hspEAsia | Japan |  |  |
| 30 | NC_017365 | F30 | hspEAsia | Japan |  |  |
| 31 | NC_017375 | 83 | hspEAsia | East Asia |  |  |
| 32 | NZ_CP011484.1 | CC33C | hspS Africa | South Africa |  |  |
| 33 | NC_017357.1 | 908 | hspWAfrica | France | West-African patient | |
| 34 | CP002332 | Gambia94/24 | hspWAfrica | Gambia |  |  |
| 35 | CP003486 | HUP-B14 | hspWAfrica | Spain |  |  |
| 36 | CP003475 | PeCan18 | hspWAfrica | Peru |  |  |
| 37 | NZ_CP011330 | J99 | hspWAfrica | USA |  |  |
| 38 | NC_017374 | 2017 | hspWAfrica | France | West-African patient | |
| 39 | NC_017381 | 2018 | hspWAfrica | France | West-African patient | |
| 40 | CP002336 | SouthAfrica7 | hpAfrica2 | South Africa |  |  |
| 41 | CP006691 | SouthAfrica20 | hpAfrica2 | South Africa |  |  |
| 42 | NZ_CP034147 | HP14039 | hpNEAfrica | Australian | Born in Somalia | |
