# Supplementary material for: The endemic Helicobacter pylori population in Southern Vietnam has both South East Asian and European origins
Source: Gut Pathog. 2021 Sep 30;13:57. doi: 10.1186/s13099-021-00452-2 (PMC8482589; doi:10.1186/s13099-021-00452-2)
Supplement: Supplementary file 2 — Additional file 2:Table S2. Sociodemographic, behavioral, clinical information of the 161 patients^ included in the study. [file 13099_2021_452_MOESM2_ESM.docx]

**S Table 2: Sociodemographic, behavioral, clinical information of the 161 patients^^^ included in the study.**

| **Characteristic** | | **Patients** | **Primary Infection** | **Secondary Infection** | ***p*-value** |
| --- | --- | --- | --- | --- | --- |
|  |  | n = 161 | n = 92 | n = 69 |  |
|  |  | n(%) | n(%) | n(%) |  |
| Age (mean; IQR) | | 39.41 (32-48) | 38.99 (32-47) | 40.17 (30-51) |  |
| Gender |  |  |  |  | 0.061 |
|  | Male | 72 (44.7) | 47 (51.09) | 25 (36.23) |  |
|  | Female | 89 (55.3) | 45 (48.91) | 44 (63.77) |  |
| Risk behavior |  |  |  |  |  |
|  | Smoking-Yes | 26 (16.1) | 16 (17.39) | 10 (14.49) | 0.145 |
|  | Alcohol-Yes | 35 (27.1) | 25 (27.17) | 10 (14.49) | 0.254 |
| Clinical symptoms | |  |  |  |  |
|  | Epigastralgia | 83 (51.6) | 49 (53.26) | 34 (49.28) | 0.683 |
|  | Abdominal Fullness | 51 (31.7) | 31 (33.7) | 20 (28.99) | 0.565 |
|  | Burping | 45 (28.0) | 25 (27.17) | 20 (28.99) | 0.8 |
|  | Acid In digestion | 37 (23.0) | 20 (21.74) | 17 (24.64) | 0.665 |
|  | Stomach Burn | 23 (14.3) | 9 (9.78) | 14 (20.29) | 0.059 |
|  | Constipation | 12 (7.5) | 7 (7.61) | 5 (7.25) | 0.931 |
|  | Diarrhea | 26 (16.1) | 16 (17.39) | 10 (14.49) | 0.621 |
| Stomach Inflammation | |  |  |  |  |
|  | Yes | 153 (95.7) | 86 (93.48) | 67 (98.55) | 0.435 |
|  | No | 8 (4.3) | 6 (6.52) | 2 (1.45) |  |
|  | One symptom | 74 (46.6) | 32 (34.78) | 42 (62.32) | 0.011* |
|  | Two symptoms | 68 (42.2) | 47 (51.09) | 21 (30.43) |  |
|  | >2 symptoms | 11 (6.8) | 7 (7.61) | 4 (5.8) |  |
| Endoscopic finding | |  |  |  |  |
|  | Erosion | 60 (37.9) | 37 (40.22) | 23 (33.33) | 0.317 |
|  | Congest | 120 (74.5) | 68 (73.91) | 52 (75.36) | 0.564 |
|  | Hemorrhage | 12 (7.5) | 7 (8.7) | 5 (7.25) | 0.571 |
|  | Edema | 42 (26.1) | 29 (31.52) | 13 (18.84) | 0.082 |
|  | Metaplasia | 8 (4.3) | 5 (5.43) | 3 (4.35) | 0.540 |

^^^Attended the Gastroenterology Department at Gia Dinh Hospital, Ho Chi Minh City, Vietnam.

^*p=>0.05^
