# Supplementary material for: The endemic Helicobacter pylori population in Southern Vietnam has both South East Asian and European origins
Source: Gut Pathog. 2021 Sep 30;13:57. doi: 10.1186/s13099-021-00452-2 (PMC8482589; doi:10.1186/s13099-021-00452-2)
Supplement: Supplementary file 3 — Additional file 3:Table S3. Putative phage regions identified in isolates using PHASTER tool and major genes encoded within these regions. [file 13099_2021_452_MOESM3_ESM.docx]

**S Table 3.** Putative phage regions identified in isolates using PHASTER tool and major genes encoded within these regions.

| Isolate | Regions | Size (kbs) | CDS | Completeness | Major functions encoded |
| --- | --- | --- | --- | --- | --- |
| GD13 | 1 | 10 | 10 | Incomplete | hypothetical protein, putative restriction-modification protein, putative thymidylate kinase, PcrA helicase |
| GD16 | 1 | 10.9 | 11 | Incomplete | hypothetical protein, putative restriction-modification protein, TMP kinase, PcrA helicase |
| GD31 | 1 | 10.9 | 12 | Incomplete | hypothetical protein, utative restriction-modification protein, TMP kinase, PcrA helicase |
| GD34 | 1 | 8.1 | 9 | Incomplete | bacteriophage DNA replication protein, DnaC homolog, glutamine amidotransferases class-II (GATase) / queuosine biosynthesis QueC ATPase, putative FAD-dependent thymidylate synthase, transposase, putative transposase, hypothetical protein, |
| GD47 | 1 | 10.9 | 11 | Incomplete | hypothetical protein, putative restriction-modification protein, TMP kinase, PcrA helicase |
| GD48 | 1 | 10.9 | 12 | Incomplete | hypothetical protein, putative restriction-modification protein, TMP kinase, PcrA helicase, |
| GD50 | 1 | 10.9 | 12 | Incomplete | hypothetical protein, putative restriction-modification protein, TMP kinase, PcrA helicase |
| GD57 | 1 | 8.5 | 7 | Incomplete | hypothetical, clamp-loader subunit, phosphoheptose isomerase, bifunctional heptose 7-phosphate kinase/heptose 1-phosphate adenyltransferase, putative nucleotide-sugar epimerase, |
| GD67 | 1 | 10.9 | 13 | Incomplete | hypothetical protein, putative restriction-modification protein, TMP kinase, PcrA helicase, |
| GD77 | 1 | 10.9 | 12 | Incomplete | hypothetical protein, putative restriction-modification protein,TMP kinase, PcrA helicase |
| GD81 | 1 | 10.9 | 11 | Incomplete | hypothetical protein, putative restriction-modification protein, TMP kinas, PcrA helicase |
| GD93 | 1 | 8.5 | 9 | Incomplete | gp245, clamp-loader subunit, phosphoheptose isomeras, bifunctional heptose 7-phosphate kinase/heptose 1-phosphate adenyltransferase, putative nucleotide-sugar epimerase, |
| GD98 | 1 | 13.9 | 14 | Incomplete | bacteriophage DNA replication protein, DnaC homolog, hypothetical protein ORF004, hypothetical protein, transposase, putative transposase, putative helicase, |
| GD99 | 1 | 8.1 | 10 | Incomplete | bacteriophage DNA replication protein, DnaC homolog, hypothetical protein ORF004, transposase, putative transposase |
